# Eating and Drinking

**Published:** 1873-06

**Authors:** 


### EATING AND DRINKING.

THE man who eats and drinks by line and plummet will always die before his time. There ought to be variety in quantity and quality every day. A man may exist on the same kind of food and the same in amount every day, for weeks and months, but it is not living—it is not a natural mode of life. What is given for the sustenance of man was intended by Infinite Beneficence to be a pleasure as well as a need. It was intended that we should have a great satisfaction in eating, and to this end there is such a diversity in the kinds of food.

So will he die before his time who eats warming food —the carbonaceous kinds—such as fats and sugars and starches, because he is thin and chilly.

The man who is stupid enough to eat "brain food," in the hope that it will give him brains, when he hadn't any before, will always fail of his object.

We all love variety in food; and variety is kindly given to us. We not only love it, but the stomach digests it; and the fact is always noticed, that whatever a man eats at an ordinary meal is converted in a short time to a mass which, in substance and color, is always alike, seeming to show that, in any ordinary meal, there are all the elements needed for the sustenance of all parts of the body, in proper proportions.

Besides this, there is given to us the instinct of appetite—to the unreasoning infant and to the unreasoning idiot; to the fool and to the philosopher alike—each following his own inclination, may prefer a different kind of food, and yet they all live on for many years.

These things go to show that to eat by rule and rate and regulation is unnatural; that a better method is to eat in moderation whatever we can get and whatever we like best; only that occasionally what we like best is followed by some discomfort, and does not "agree" with us; the reason of this sometimes being that we eat too much of it. In such a case, it is better to eat less and less every day, until we find out how much we can take with impunity. But a thing may not agree with us to-day, even in small quantities, yet may do so a week or a month or a year hence.

Besides all this, people who are all the time thinking about what they are going to eat, either haven't much sense, or are more animals than men; and if they were only compelled to earn their own living by some out-door employment, would be very glad to get anything to eat, and would soon be able to eat anything, and no questions asked.

